# Unveiling the ecotoxicological effects of azoxystrobin-based fungicides at realistic concentrations on the land snail, *Theba pisana*

**DOI:** 10.1038/s41598-024-66416-z

**Published:** 2024-07-16

**Authors:** Mohamed A. Radwan, Amira F. Gad, Amira M. Abd El-Aziz, Kawther S. El-Gendy

**Affiliations:** 1 Department of Pesticide Chemistry and Technology, Faculty of Agriculture, University of Alexandria, El-Shatby 21545, Alexandria, Egypt; 2 Department of Animal Pests, Plant Protection Research Institute, Agricultural Research Center, Alexandria, Egypt

**Keywords:** Land snails, Azoxystrobin, Physiology, Oxidative stress, Neurotoxicity, Histopathology, Biochemistry, Biotechnology, Physiology, Environmental sciences, Biomarkers

## Abstract

The ecotoxicological consequences of azoxystrobin on land snails have not yet been addressed. Therefore, the present study aims to provide novel data on the threat of a commercial grade azoxystrobin (AMISTAR) at two environmentally relevant concentrations (0.3 µg/ml) and tenfold (3 µg/ml) on the model species, *Theba* *pisana* by physiological, biochemical, and histopathological markers for 28 days. Our results showed a reduction in animal food consumption and growth due to exposure to both azoxystrobin concentrations. It also induced oxidative stress and led to a significant decrease in lipid peroxidation (LPO) levels after 7 days of exposure, while the opposite effect occurred after 28 days. Except for the 7-day exposure, all treated snails had significantly reduced glutathione (GSH) content and increased catalase (CAT) activity at all-time intervals. Glutathione peroxidase (GPx), glutathione-S-transferase (GST) activities, and protein content (PC) were elevated in treated snails at all-time intervals. Moreover, alterations in acetylcholinesterase (AChE) activity between a decrease and an increase were noticed. Additionally, azoxystrobin exerted changes in *T. pisana* hepatopancreas architecture. Our study suggests that azoxystrobin may have negative ecological consequences for *T. pisana* and highlights its potential risks to the natural environment.

## Introduction

Environmental pollution is increasing due to anthropogenic activities, including pesticides. Organic pesticides are widely applied to control various pests to ensure food security on a global scale. Their residues can contaminate the environment and generate serious concerns about public health in addition to ecological imbalance^1^. Among them, azoxystrobin fungicide can pose a threat to the natural environment by promoting the accumulation and migration of toxic substances in water and soil^2^ and its potentially disastrous ecological effects are crucial to the health of the environment.

Azoxystrobin is a broad-spectrum systemic strobilurin fungicide^3^. It has a toxic action on fungi by blocking the electron transport chain in mitochondria, resulting in the prevention of ATP production and increasing oxidative damage^4,5^. It has low volatility, low mobility in soil, and low to moderate persistence in the environment^6,7^*.* Its dissipation in soil occurs mainly due to photolysis and microbial degradation. The upper layers of the soil (0–20 cm) showed a greater residue level and limited mobility for several azoxystrobin formulations^8^. European soils currently contain up to 0.25 mg a.i./kg of azoxystrobin^9^. In Chinese ginseng fields, its initial residue was 9.54 mg/kg in the fields of Jilin Province and 8.57 mg/kg in Beijing Province^10^. It is regularly found in foods^11^. In addition, azoxystrobin has been detected in aquatic and terrestrial ecosystems at concentrations exceeding Regulatory Acceptable Concentrations (RACs) and may threaten non-target creatures^5^. It is classified as having a moderate risk to earthworms and bees, but a high risk to fish and aquatic crustaceans^6,7,11^. It causes oxidative stress in earthworms, *Eisenia fetida*^12^, crayfish^13^, and male rats^14^.

To determine the true impact on non-target organisms, testing a commercial pesticide product actually applied to the environment is essential since the toxicity of the product is often greater than that of the active ingredient alone^15^. Excipients and an active ingredient are the two main components of pesticide formulations. Despite being considered inert chemicals, the toxicological characteristics of excipients may even more significant than those of the active ingredient and should not be ignored^16^. Their presence in commercial formulations may increase the toxicity of pesticides, either due to their toxic properties or by favouring the bioavailability of the active ingredient^17^. As a result, assessing the toxicity of commercial formulations is a more realistic approach to the effects of pesticides on non-target organisms^15^.

In the terrestrial environment, a rising increase in contaminants and their residues has been noted over time. In this context, the necessity of developing better tools to identify and assess the ecotoxicological effects of chemicals is emphasized^18^. The use of bioindicators in ecotoxicological research directly helps determine the risk of these residues really existing in the terrestrial environment because chemical monitoring is becoming less instructive regarding ecological repercussions^19^.

Land snails are frequently employed as bioindicator organisms used in risk assessment as they are characterized by their large size, limited mobility, long-life span, and being easy to identify and collect. They are capable of being fed artificial diets, responding quickly to toxicants at sublethal levels, and accumulating toxic substances^20^. The terrestrial *Theba pisana* species is widespread throughout the world and is the most prevalent in the Mediterranean region. It has gained interest as a potential sentinel species used to signal chemical contamination of terrestrial ecosystem^21,22^.

When subjected to xenobiotic stress, living organisms adapt by transforming into new physiological states that preserve homeostasis. Stress can cause cellular damage and trigger the activation of repair systems if its effects surpass homeostasis^23^. To counteract these impacts, biomarkers are altered to address these stresses. Biomarkers are a sensitive tool to evaluate stress caused by xenobiotics^24^. They can assess biological entities at multiple levels, including molecules, cells, individuals, and populations, which endows them with an integrative character about the whole sequence of events that ensues after being exposed to pollution^25^. Physiological, biochemical, and histopathological indicators are the most suitable diagnostic tools and economical approaches for the early-warning detection of environmental contaminants^26^. Oxidative stress biomarkers can be used to measure the association between adverse impacts and pollutant exposure. This refers to the reactive-oxygen-species (ROS) generation that results in damage to cellular and tissue components, processes, structures, or functions^27^.

Although the biomarker responses of land snails subjected to different contaminants, including pesticides, has recently been extensively evaluated^28,29^, the ecotoxicological impact of azoxystrobin on land snails have not yet been addressed. So, the goal of the present study was to ascertain for the first time the effects of a commercial grade azoxystrobin at both environmental concentrations (0.3 µg/ml) and tenfold (3 µg/ml) exposure for 28 days on the model species, *T. pisana*. This study specifically aims to critically assess the following topics: (1) Effect of azoxystrobin on the physiological response of snails (e.g., feeding behaviour and growth), (2) Biochemical effects using biomarkers such as catalase (CAT), lipid peroxidation (LPO), reduced glutathione (GSH), glutathione peroxidase (GPx), glutathione S-transferase (GST) and a neurotoxic biomarker, acetylcholinesterase (AChE) and protein content (PC) after 7, 14, 21, and 28 days of exposure, (3) Histopathological examinations in the hepatopancreas of *T. pisana* snail's post-28 days of exposure.

## Materials and methods

### Test chemicals

The commercial azoxystrobin (AMISTAR 25% SC) with molecular formula (C_22_H_17_N_3_O_5_) used in this study was provided by Syngenta Agro Services AG, Egypt. All chemicals and reagents utilized were from Sigma-Aldrich.

### Test snails

The land snail, *Theba pisana* was utilized as the test animal. Healthy adult specimens with shell diameters of 18.1 ± 0.067 mm and body weights of 0.93 ± 0.04 g were gathered from an uncontaminated garden (Antoniades) in Alexandria, Egypt. Prior to the trials, the gathered snails were acclimatized for at least 14 days in aerating cages (45 × 50 × 50 cm, with 150 individuals per cage) under laboratory conditions (26 ± 2 °C and 65 ± 2 RH), with a 12:12 light: dark photoperiod, fed lettuce leaves, and starved for 48 h before treatment. All experimental methods were performed in accordance with the ethical animal guidelines and regulations set by the Alexandria University Ethics Committee (protocol number:AU082209201105). This study was carried out in compliance with the ARRIVE guidelines.

### Diet preparation and experimental design

The artificial diet was prepared by mixing 5 g rabbit meal, 3 g sucrose, and 2 g agar with 100 ml of water^30^. An aliquot of azoxystrobin stock solution was mixed with agar medium to obtain the two tested environmentally relevant concentrations (0.3 and 3 µg/ml). The obtained medium was equally distributed over four Petri dishes. Petri dishes were chilled before being stored in the fridge. The artificial diet was cut into discs (each 3-cm in diameter) using a stainless-steel cork borer and offered to snails. Snails were exposed to an azoxystrobin-contaminated diet for 7, 14, 21, and 28 days of treatment to evaluate its effects on physiological and biochemical parameters of *T. pisana* snails.

### Effect of the environmental azoxystrobin concentrations on some biomarkers of *T. pisana* snails

Two environmentally relevant azoxystrobin concentrations (0.3 and 3 μg/ml) were selected based on its ecotoxicological profile to reflect environmental realities^9–11^. In the experiment, 180 snails were divided into 3 groups (60 individuals per group, each 3 repetitions); the first group served as the control and was given a non-toxic food. The snails in the second group received food treated with 0.3 µg/ml azoxystrobin, and the third group received food treated with 3 µg/ml azoxystrobin.

### Physiological parameters

#### Feeding and growth responses

A treated diet containing tested concentrations of azoxystrobin was provided to the snails in 12-cm-diameter plastic boxes. The boxes were kept under laboratory conditions, and the animals were kept moist by sprinkling with water every day. Snails were given fresh agar discs to eat during the experiment. Boxes were checked daily to remove the dead snails. Fresh agar discs were weighed on a regular basis and provided to snails as needed. The remaining uneaten diet was collected, cleaned of excrement, and dried at 60 °C until it reached a constant weight. The boxes were cleaned, and the snails were weighed weekly for up to four weeks. The mean weight (g) of dry food ingested per animal per time interval was used to calculate the feeding rate. According to Gomot^31^, the snail mass was determined by weighing each animal at the beginning of the experiment and again after 7, 14, 21, and 28 days in order to calculate the change in animal mass (g).

### Oxidative stress parameters

#### Sample preparation

Following 7, 14, 21, and 28 days of treatment, the two organs (head-foot and digestive gland) of the survivor snails from each group were rapidly removed, rinsed, and cleared of any extra connective adipose tissues. Each organ was independently weighed and homogenized with 0.9% cold saline in 10 volumes for 60 s in a polytron homogenizer (Tekmar tissumizer). A Janetzki K 23 cooling centrifuge was used to centrifuge the homogenate of each organ at 3000×*g* for 30 min at 4 °C. LPO and GSH levels were measured in the homogenate of hepatopanceas. CAT, GPx, GST activities, and PC were estimated in the hepatopanceas supernatant, while AChE activity was assessed in the head-foot supernatant.

### Non-enzymatic parameters

### LPO level

Malondialdehyde (MDA) production was quantified using the thiobarbituric acid (TBA) technique established by Placer et al.^32^. The level of LPO is denoted as nmoles MDA/g wet tissue.

#### GSH content

Using the method outlined by Owens and Belcher^33^, the content of GSH was measured at 412 nm. After calibration using the standard GSH curve, its content was presented as mg GSH /g wet tissue.

### Enzymatic parameters

#### CAT activity

The activity of CAT was measured at 240 nm using Beers and Sizer^34^ method. It is represented in unit/g wet tissue.

#### GPx activity

GPx activity was assessed based on Chiu et al.^35^. The enzyme activity was measured at 412 nm and displayed as nmoles/mg protein.

#### GST activity

The activity of GST was estimated using the method outlined by Vessey and Boyer^36^. Its activity was represented as μmole/min/mg protein.

### Neurotoxic biomarker

#### AChE activity

The method used for measuring AChE activity has been described by Ellman et al.^37^. The assay was performed using the substrate, acetylthiocholine iodide. The AChE activity was presented as μmole/min/mg protein.

### PC

Using bovine serum albumin (BSA) as a standard, protein content was measured in accordance with Lowry et al.^38^.

### Histopathological evaluations

At the end of the experiment (28 days), the digestive glands of three snails from either the control or those exposed to the tested concentrations of azoxystrobin were dissected. The digestive glands that had been dissected were preserved for a minimum of 24 h in 10% formalin. Overnight, the fixative solution was washed under running water. The fixed tissues were dried with alcohol, embedded in paraffin wax at 56 °C in an oven, sectioned (4 μm thickness), mounted on glass slides, deparaffinized, and stained with hematoxylin and eosin^39^. The cytological examinations were conducted under 40× magnification using a Leica DM500 optical microscope, Heerbrugg, Switzerland, and photomicrographs were taken in a bright field.

### Data analysis

To ensure normality and homogeneity of variance, all the data were statistically examined using the Shapiro–Wilk and Levene's tests, respectively. The data obtained were represented as the mean ± standard deviation (SD). The ANOVA analysis of data was followed by the separation of the means using the Student–Newman–Keuls test at a probability level (*p* ≤ 0.05). Costat Software Version 2.6^40^ was used for statistical analysis.

### Ethical declarations

This study was carried out in compliance with the ARRIVE guidelines.

## Results

### Physiological biomarkers

#### Feeding behaviour

**Figure 1:**
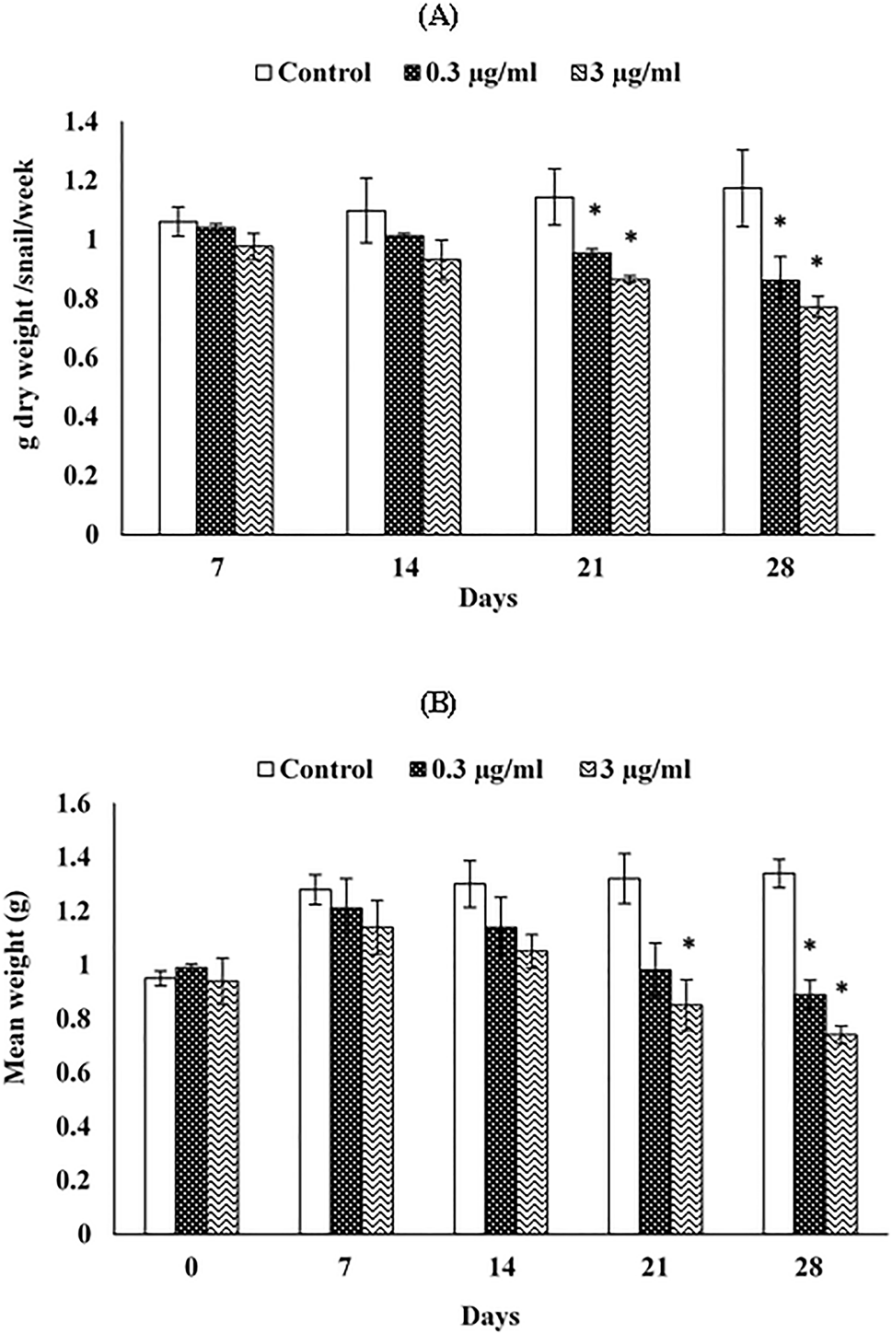
Food consumption (**A**) and growth rate (**B**) of *Theba pisana* snails exposed to contaminated food with two realistic azoxystrobin concentrations at various time intervals. Values are mean ± standard deviation, n = 3. * Significantly different from the control value (*p* ≤ 0.05).

Dietary azoxystrobin exposure, at the two tested concentrations of 0.3 and 3 µg/ml, significantly reduced the food consumption of snails after 21 and 28 days compared with untreated snails. After 21 days of exposure, the control group had ingested 1.14 g dry food, whereas this amount dropped to 0.95 and 0.86 g dry food for snails treated with 0.3 µg/ml and 3 µg/ml azoxystrobin, respectively. The food consumption was 1.17 g dry food in the control group, while it was 0.86 and 0.77 g dry food for snails treated with low and high azoxystrobin concentrations, respectively, after 28 days of exposure (Fig. 1A).

### Growth responses

The growth rate of snails decreased with increasing time of exposure in a manner similar to food consumption. The growth of treated animals was slightly increased from the beginning of the trial up to 14 days, and then gradually decreased. After 21 days of exposure, a marked decline in the growth of snails subjected to 3 µg/ml azoxystrobin was observed. Likewise, significant decreases in growth of intoxicated snails with the two tested concentrations of azoxystrobin were noted after 28 days of exposure, where the mean weight value was 0.89 g when subjected to a 0.3 µg/ml azoxystrobin-contaminated diet and 0.74 g for 3 µg/ml azoxystrobin-contaminated diet in comparison with 1.34 g for untreated snails (Fig. 1B).

### Oxidative stress biomarkers

#### LPO level

The obtained results clearly indicate that LPO levels in treated snails with azoxystrobin for 7 days of exposure were significantly reduced to 84.54 and 85.45% of control at concentrations of 0.3 and 3 µg/ml, respectively. However, an insignificant decrease or increase in LPO was observed in snails treated with azoxystrobin concentrations after 14 and 21 days of exposure. At the end of the experiment (28 days), both tested azoxystrobin concentrations caused a significant increase in LPO level compared to the control (Table 1).

**Table 1:** Lipid peroxidation (LPO) level in the hepatopancreas of *Theba pisana* exposed to the two relevant azoxystrobin concentrations at different time intervals.

Days after exposure	LPO level (nmoles of MDA/mg wet tissue)				
	Control	0.3 µg/ml		3 µg/ml	
	Mean ± SD	Mean ± SD	% of Control	Mean ± SD	% of Control
**7**	1.10** ± **0.003^a^	0.93** ± **0.07^b^	84.54	0.94** ± **0.09^b^	85.45
**14**	1.00** ± **0.03^a^	0.90** ± **0.19^a^	90.00	0.98** ± **0.03^a^	98.00
**21**	1.22** ± **0.03^a^	1.18** ± **0.05^a^	96.72	1.24** ± **0.20^a^	101.63
**28**	1.20** ± **0.03^a^	1.41** ± **0.03^b^	117.50	1.85** ± **0.07^c^	154.16

Mean followed by the same letter(s) in each row are not significantly differences from control (*p* ≤ 0.05).

#### GSH content

Except for 7 days of exposure, the GSH level was markedly decreased in snails exposed to 0.3 µg/ml azoxystrobin by 89.32, 89.22, and 88.98% and by 87.30, 77.61, and 91.18% for 3 µg/ml azoxystrobin, after 14, 21, and 28 days, respectively (Table 2).

**Table 2:** Reduced glutathione (GSH) content in the hepatopancreas of *Theba pisana* exposed to the two relevant azoxystrobin concentrations at different time intervals.

Days after exposure	GSH content (mg GSH/g. wet tissue)				
	Control	0.3 µg/ml		3 µg/ml	
	Mean ± SD	Mean ± SD	% of Control	Mean ± SD	% of Control
7	6.26 ± 0.57^a^	5.73 ± 0.15^a^	91.53	5.62 ± 0.17^a^	89.77
14	6.93 ± 0.05^a^	6.19 ± 0.27^b^	89.32	6.05 ± 0.26^b^	87.30
21	6.03 ± 0.24^a^	5.38 ± 0.02^b^	89.22	4.68 ± 0.02^b^	77.61
28	5.90 ± 0.05^a^	5.25 ± 0.31^b^	88.98	5.38 ± 0.10^b^	91.18

Mean followed by the same letter(s) in each row are not significantly differences from control (*p* ≤ 0.05).

#### CAT activity

Results clearly indicated that, after 7 days of exposure, the mean CAT activity value was markedly enhanced in snails subjected to 0.3 µg/ml azoxystrobin. The CAT activity was elevated in snails treated with the two concentrations of azoxystrobin after 14, 21, and 28 days of exposure (Table 3).

**Table 3:** Effect of the two realistic azoxystrobin concentrations on catalase (CAT) activity of *Theba pisana* after different times of exposure.

Days after exposure	CAT activity (Units/g. tissue)				
	Control	0.3 µg/ml		3 µg/ml	
	Mean ± SD	Mean ± SD	% of Control	Mean ± SD	% of Control
7	177.83** ± **11.76^a^	235.33** ± **20.29^b^	132.33	154.34** ± **2.69^ab^	86.79
14	197.72** ± **7.64^a^	251.75** ± **3.18^b^	127.32	238.31** ± **25.41^b^	120.52
21	184.98** ± **1.24^a^	229.92** ± **33.40^c^	124.29	210.34** ± **5.85^b^	113.70
28	169.10** ± **17.85^a^	200.01** ± **12.99^b^	118.27	180.65** ± **8.98^a^	106.83

Mean followed by the same letter(s) in each row are not significantly differences from control (*p* ≤ 0.05).

#### GPx activity

Except for 7 days of exposure, a significant increment in GPx activity was observed in snails exposed to 0.3 µg/ml azoxystrobin after 14, 21, and 28 days. On the other hand, azoxystrobin at 3 µg/ml caused a significant enhancement in GPx activity after 7, 14, and 21 days while no significant increase was observed after 28 days of exposure (Table 4).

**Table 4:** Effect of the two realistic azoxystrobin concentrations on glutathione peroxidase (GPx) activity of *Theba pisana* after different times of exposure.

Days after exposure	GPx activity (nmoles/mg protein)				
	Control	0.3 µg/ml		3 µg/ml	
	Mean ± SD	Mean ± SD	% of Control	Mean ± SD	% of Control
7	95.20 ± 2.46^a^	112.44 ± 7.52^ab^	118.10	121.60 ± 0.52^b^	127.73
14	96.36 ± 5.88^a^	117.82 ± 17.14^b^	122.27	131.17 ± 1.45^b^	136.12
21	129.30 ± 1.40^a^	165.30 ± 5.43^b^	127.84	190.93 ± 1.30^b^	147.66
28	112.62 ± 1.38^a^	136.36 ± 5.43^b^	121.07	115.14 ± 6.45^a^	102.23

Mean followed by the same letter(s) in each row are not significantly differences from control (*p* ≤ 0.05).

#### GST activity

Snails exposed to both azoxystrobin concentrations had significant increases in GST activity compared to controls, except after 28 days of exposure. Induction of GST in treated snails with the concentration of 0.3 µg/ml azoxystrobin was recorded as 1.39, 1.28, 1.15, and 1.09-fold of control and as 1.35, 1.15, 1.12, and 1.08 in treated snails with a concentration of 3 µg/ml azoxystrobin for 7, 14, 21 and 28 days, respectively (Table 5).

**Table 5:** Effect of the two realistic azoxystrobin concentrations on glutathione-S-transferase (GST) activity of *Theba pisana* after different times of exposure.

Days after exposure	GST activity (µmoles/min/mg protein)				
	Control	0.3 µg/ml		3 µg/ml	
	Mean ± SD	Mean ± SD	% of Control	Mean ± SD	% of Control
**7**	0.385** ± **0.07^a^	0.538** ± **0.03^b^	139.47	0.521** ± **0.12^b^	135.32
14	0.728** ± **0.10^a^	0.933** ± **0.02^c^	128.15	0.841** ± **0.02^b^	115.52
21	0.830** ± **0.12^a^	0.960** ± **0.14^c^	115.66	0.930** ± **0.15^b^	112.04
28	0.787** ± **0.07^a^	0.865** ± **0.03^a^	109.91	0.850** ± **0.07^a^	108.00

Mean followed by the same letter(s) in each row are not significantly differences from control (*p* ≤ 0.05).

### Neurotoxic biomarker

#### AChE activity

**Figure 2:**
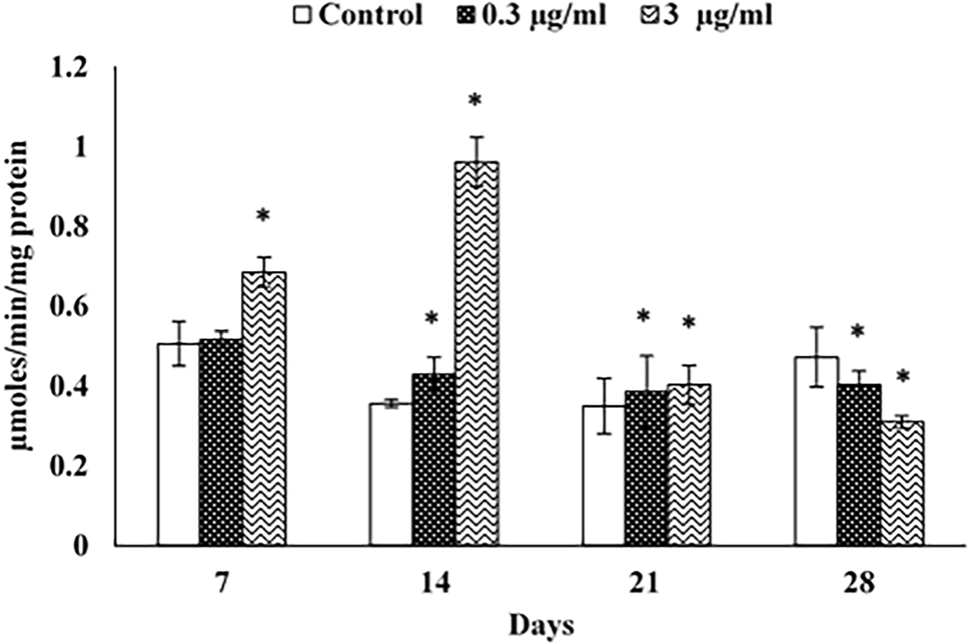
Acetylcholinesterase activity (AChE) in the head-foot of *Theba pisana* exposed to the two realistic azoxystrobin concentrations at different time intervals. Values are mean ± standard deviation, n = 3. * Significantly different from the control value (*p* ≤ 0.05).

AChE activity was non-significantly augmented in snails treated with azoxystrobin at 0.3 µg/ml, while significantly elevated in snails subjected to 3 µg/ml azoxystrobin after 7 days of exposure compared to control. After 14 and 21 days, both azoxystrobin concentrations significantly enhanced AChE activity in the animal. However, a significant inhibition of AChE activity was noticed after 28 days of exposure, as the inhibition value was 85.20% for a concentration of 0.3 µg/ml and 65.53% for 3 µg/ml (Fig. 2).

#### PC

**Figure 3:**
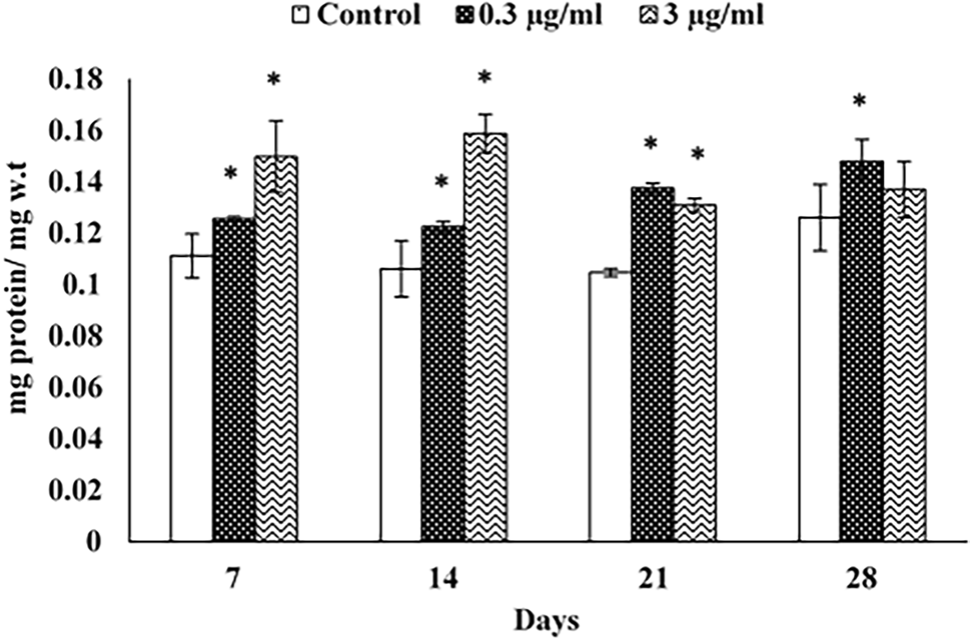
Protein content (PC) in the hepatopancreas of *Theba pisana* exposed to the two realistic azoxystrobin concentrations at different time intervals. Values are mean ± standard deviation, n = 3. * Significantly different from the control value (*p* ≤ 0.05).

Protein content in snails treated with 0.3 µg/ml azoxystrobin was significantly higher than that of untreated snails after all time intervals. This induction was recorded as 1.12, 1.15, 1.31, and 1.17 folds of control after 7, 14, 21, and 28 d, respectively. Treatment with azoxystrobin at 3 µg/ml resulted in a significant increase in protein content compared to the control, except after 28 d of exposure. This elevation was recorded as 134.23, 149.05, 125.00, and 108.73% after 7, 14, 21 and 28 d, respectively (Fig. 3).

#### Histopathological evaluations

**Figure 4:**
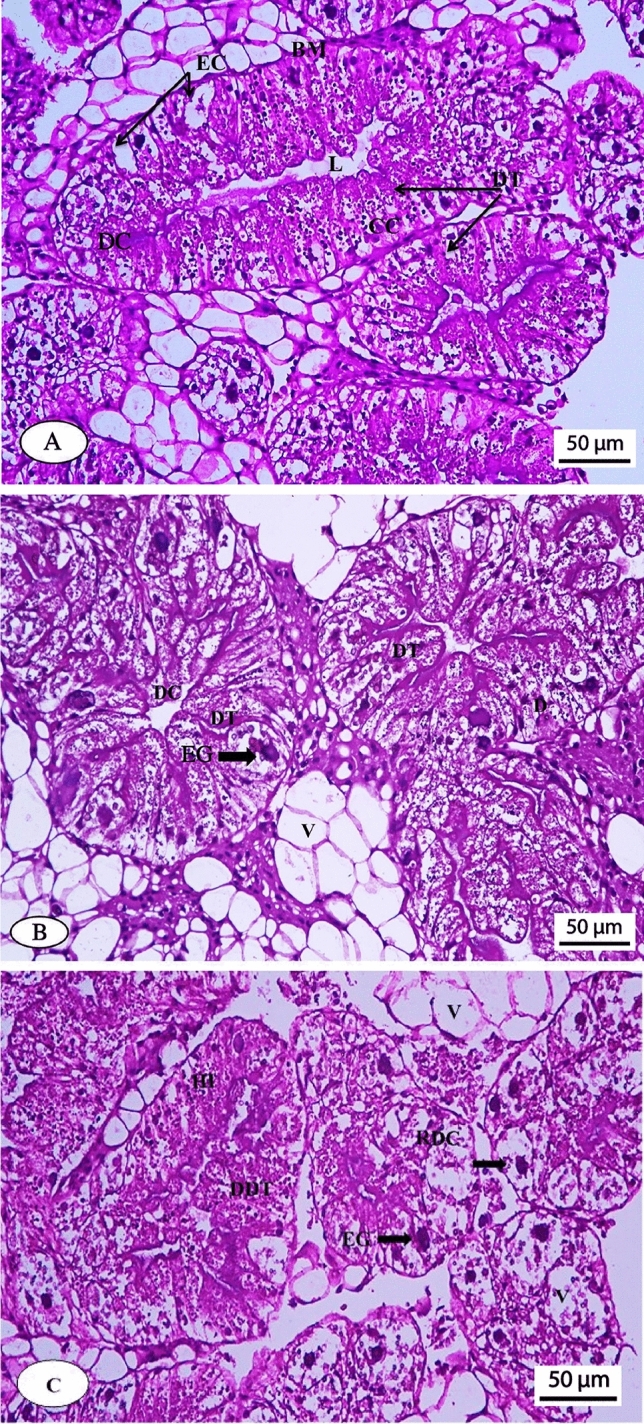
Photomicrograph of the normal hepatopancreas of *Theba pisana* snail (A), hepatopancreas of the snail treated with 0.3 µg/ml azoxystrobin (B) and hepatopancreas of the snail treated with 3 µg/ml azoxystrobin (C). Scale bar (A, B and C): 50 μm. DC, digestive cells; DT, digestive tubules; EC, excretory cells; L, lumen; C, calcium cells; BM, bilayer membrane; V, vacuoles; HI, hemocyte infiltration; EG, excretory granules; DDT, destructed digestive tubules; RDC, ruptured digestive cells.

Figure 4A, B, and C shows the photomicrographs of hepatopancreas of untreated and azoxystrobin-treated snails.

#### Untreated group

The digestive cells (DC), digestive tubules (DT), excretory cells (EC), lumen (L), calcium cells (C), and bilayer membrane (BM) of the untreated snails had a normal manifestation, showing no histopathological alterations (Fig. 4A).

#### Treated groups

A photomicrograph of the hepatopancreas of *T. pisana* snails subjected to azoxystrobin at 0.3 µg/ml showed filtration between digestive tubules; digestive cells and vacuoles (V); and excretory cells with numerous excretory granules (EG) of variable size (Fig. 4B).

The hepatopancreas of *T. pisana* snails subjected to azoxystrobin at 3 µg/ml exhibited destructed digestive tubules (DDT); ruptured digestive cells (RDC); hemocyte infiltration (HI); increased vacuoles, and the presence of excretory cells with excretory granules of variable size and clumps **(**Fig. 4C). Based on the above-mentioned symptoms, a high concentration of azoxystrobin appeared to be more toxic to the snail hepatopancreas than a low one.

## Discussion

Although azoxystrobin is recognized as a reduced-risk pesticide^41^, concerns have recently arisen about the toxicity of its formulation to non-target organisms^15^. Many researchers have found that azoxystrobin is toxic to both target fungi and non-target creatures inhabiting terrestrial and aquatic ecosystems. Yet, there have been no reports so far regarding the negative effects of azoxystrobin on non-target terrestrial snails. In this study, we sought to determine whether the azoxystrobin formulation, when used at environmentally relevant concentrations, has deleterious effects on the physiology of *T. pisana*, as measured by physiological disturbances, biochemical and histopathological changes in the animals.

Exposure to xenobiotics generally changes the behaviour of organisms, so physiological endpoints are essential to understanding the impacts of pollutants on species fitness and their physiology in a natural environment^42^. Feeding and growth are sensitive early warning indicators, as other physiological measures for most pollutants exist in ecosystems even at low concentrations^43^.

Feeding behaviour is among the physiological endpoints that have environmental significance^43^. In land gastropods, the central nervous system controls the feeding behaviour^44^. Effects on organisms' feeding can then be translated into impacts on growth and subsequent on populations^45^.

Our results indicate that snails' food consumption decreases when exposed to two realistic azoxystrobin concentrations, suggesting treated snails experience physiological stress. These decreases in food intake may be a consequence of the neurotoxic effect of azoxystrobin, which changes the feeding behaviour of intoxicated snails. The impact of azoxystrobin on the food intake of land snails has not been examined so far, even though the toxic effects of several pesticides on food consumption have been studied. For instance, when *Helix aspersa* snails were treated with carbaryl, fenitrothion, azinphos methyl, methyl parathion, aminocarb, trichlorfon, and paraquat in a 10-day feeding test, they exhibited significant decreases in their food consumption rate^46^. The food consumption of *H. aspersa* subjected to a pentachlorophenol-contaminated diet was sharply decreased^20^. Furthermore, significant decreases in the food consumption rate of *T. pisana* were noticed after 14 days of exposure to abamectin, thiamethoxam, and acrylamide^47^.

Growth is another fundamental physiological indicator of population survival^48^. In the current work, the decrease in growth of azoxystrobin-treated animals might be a result of the chemical interference in their feeding activity. It's feasible that decreases in snail growth correspond to decreases in the food consumption rate.

To our knowledge, the azoxystrobin impact on land snails' growth has not been documented in any published literature. Nevertheless, the present data fully align with the observation of Coeurdassier et al.^49^ who found that the growth parameters of *H. aspersa* decreased after 28 days of exposure to dimethoate. Moreover, there were significant decreases in the shell diameter and total weight of *H. aspersa* snails treated with carbaryl, fenitrothion, azinphos methyl, methyl parathion, aminocarb, trichlorfon, and paraquat in the 14 days feeding test^46^. Indeed, after 14 days of dietary treatment with abamectin and thiamethoxam, the growth of *T. pisana* decreased^47^^.^

An imbalance between pro-oxidants and antioxidants is known as oxidative stress, which is caused by elevated ROS production and/or a depleted antioxidant defense system. When the balance between ROS production and detoxification system ability is disturbed, ROS accumulates inside the cell and causes adverse impacts^50^. Some invertebrate species have been shown to have increased oxidative stress as one of the negative impacts of azoxystrobin^12^ and there is no data regarding land snails.

The free radicals assault the cellular membrane, starting a process known as LPO, which is an autocatalytic oxidation of polyunsaturated fatty acids (PUFAs)^50^. Malondialdehyde (MDA), the end product of LPO, is utilized as a diagnostic tool for tissue injury and oxidative damage in gastropods^51^.

The increased levels of LPO in the treated snails in our study may be attributed to either an excess of ROS or a decrease in the activity of the antioxidant system, implying the occurrence of oxidative damage and an insult to the integrity of the cell's structure^52^. On the other hand, we observed a reduction in LPO levels which could be due to the antioxidant system's protective function in lowering LPO levels caused by ROS.

The obtained outcomes are consistent with Ali et al.^51^ who observed that, in the digestive gland of *Lymnaea luteola* freshwater snail, LPO level significantly increased post-exposure to 1/10, 1/2, and 2/3 LC_50_ of azoxystrobin for 24 and 96 h. Also, Xu et al.^53^ found that treatment of earthworm, *E. fetida* with azoxystrobin at 0, 0.1, 1.0 and 2.5 mg/kg on the 7, 14.21, 28, 42, and 56 days resulted in increases in LPO levels in three types of soil. Moreover, *E. fetida* exposed to azoxystrobin at 0.1 and 10 mg/kg exhibited a significant decrease in LPO level after 7 days, while showing an increase in LPO level after 14, 21, and 28 days^12^.

GSH is an essential conjugate of electrophiles in detoxifying mechanisms, co-substrates in a number of antioxidant enzymes such as GST and GPx, and prevents oxidative injury via scavenging oxygen radicals^54^.

According to our data, reduced GSH content in *T. pisana* exposed to azoxystrobin is often linked to the acceleration of peroxidation processes in the cell membrane, a disruption in the GSH production pathway^55^ or consumption of GSH that is associated with an increase in GPx and GST activities^56^. The results obtained are consistent with data published by Vieira et al.^57^ who found a significant decrease in GSH level in larvae of zebrafish after intoxication with concentrations of 1, 10, and 100 μg/L azoxystrobin**.** Similarly, Ali et al.^51^ showed that sub-lethal exposure to azoxystrobin decreased the level of GSH in* L. luteola* snails.

Hematin-containing CAT is a primary antioxidant enzyme that prevents cellular oxidative damage via the conversion of hydrogen peroxide (H_2_O_2_) into O_2_ and H_2_O. In the current work, increased CAT activity in snails subjected to azoxystrobin presumably results from an increase in ROS production, which elevates CAT activity in response to this stress and protects cells from oxidative damage.

CAT activity changes in molluscs have been widely utilized as a marker in biomonitoring investigations^58^. In this context, various reports have been published in the literature; in three soil types, CAT activity was enhanced in *E. fetida* earthworms subjected to azoxystrobin at 0.1, 1.0, and 2.5 mg/kg on 7, 14, 21, 28, 42, and 56 days after treatment^53^. Moreover, Kovačević et al.^15^ reported that azoxystrobin caused an elevation in the CAT activity of the annelid *Enchytraeus albidus* over 7 and 21days of exposure.

GPx is a universal enzyme found in the cytoplasm of cells and is one of the major defense enzymes for protecting creatures from damage caused by oxyradical production. It is the most significant peroxidase involved in detoxifying H_2_O_2_ to O_2_^59^.

In our study, increases in GPx activity of snails exposed to dietary azoxystrobin can be linked with an increase in ROS, which in turn can stimulate the GPx activity to scavenge peroxides. Furthermore, the lack of discernible alterations in GPx activity noticed in this investigation implies that cellular defense mechanisms effectively counteract pesticide toxicity. The results obtained coincide with the findings published by Vieira et al.^57^ who noticed no changes in the activity of GPx in larvae of zebrafish after intoxication with concentration of 1, 10, and 100 μg/L azoxystrobin**.**

A significant phase II enzyme, GST, is essential in the detoxification process to protect cells from chemical-induced oxidative stress. This enzyme neutralizes the electrophilic sites of a wide range of electrophiles by conjugating reduced GSH with them and making the compounds more water-soluble^60^.

The current study suggests that the elevation in GST activity in snails exposed to azoxystrobin may be caused by the pollutants' stimulation of the natural antioxidant defense mechanism. Also, an augmentation of GST activity is observed in response to xenobiotics which arises from the detoxification of the xenobiotics, by glutathione conjugate formation^61^.

These results are in conformity with Han et al.^12^ who showed that chronic exposure of *E. fetida* to three azoxystrobin concentrations (0.1, 1, and 10 mg/kg) revealed a significant increase in GST activity after 7, 14, 21, and 28 days. Furthermore, azoxystrobin mostly activated GST activity in earthworms in the three distinct natural soils (fluvo-aquic soil, black soil, and red clay soil), as well as an artificial soil, except for the 14, 21, and 42 days in the red clay soil, where there were no noticeable dose-toxic impacts^53^. Also, Ali et al.^51^ found an increase in GST activity of the freshwater snail**,** *L. luteola* exposed to azoxystrobin at concentrations of 0.079, 0.40, and 0.53 mg/l for 24 and 48 h except when exposed to 0.40 mg/l for 24 h. Vieira et al.^57^ showed that the concentration of 1 µg/l azoxystrobin caused an increase in GST activity in zebrafish following exposure for 96 h. Inductions in the activity of GST were noted in *E. albidus*, was exposed to azoxystrobin for 7 and 21 days ^15^.

AChE is a substantial enzyme in the nervous system for maintaining optimal behaviour and muscle function via hydrolyzing acetylcholine, which acts as a neurotransmitter (ACh), to acetate and choline, thus terminating synaptic transmission occurred^62^. AChE is excessively utilized as a dependable neurological marker for a wide range of environmental pollutants, particularly organophosphate and carbamate pesticides^63^.

The two azoxystrobin concentrations that were examined in our study increased AChE activity during some exposure intervals, which may have had a detrimental effect on *T. pisana* snail's neurological system. These findings suggest that oxidative damage shown as LPO in azoxystrobin-treated snails was the cause of elevated AChE activity. It was observed in a related investigation that AChE activation in the rat brain was linked to a decline in the antioxidant state^64^. In other exposure intervals, however, azoxystrobin exhibited a neurotoxic impact in exposed snails, as shown by the suppression of AChE activity. The inhibitory mechanisms might indicate that the tested substance prohibits nerve impulse transmission throughout the nervous system. Because AChE is unable to hydrolyze ACh, accumulation of ACh may occur, which may cause hyperstimulation, a loss of muscular control, and eventually death^62^. Furthermore, the indirect impact of ROS, especially H_2_O_2_ produced after exposure to pesticides, may also be associated with the suppression of AChE. In fact, the amount of H_2_O_2_ produced by the LPO pathway regulates the activity of AChE and modifies the enzyme active site^65^.

The obtained data are comparable to those obtained by Vieira et al.^57^ who showed that exposure of zebrafish larvae to 1 μg/l azoxystrobin for 96 h significantly elevated AChE activity compared to the controls.

Protein is a large molecule included in the architecture, metabolism, and physiology of cells. It also plays a key role in DNA replication and the transportation of molecules from one site to another.

Under stress, the animal has the capability to produce stress proteins. Given this, an increase in protein content in azoxystrobin-treated snails may be due to the snails needing more energy to cope with the induced stress and detoxify the toxicant. The obtained results contradict those reported by Liu et al.^66^ who showed that azoxystrobin treatments markedly reduced the soluble protein content in *Chlorella vulgaris* after 48 and 96 h.

The toxic impact of xenobiotics on an organism could be assessed by observing the histopathological alterations in different tissues and organ^67^. Gastropods' hepatopancreas is recognized to be a major target organ for xenobiotics. Any architectural injury to the hepatopancreas has an impact on the animals in a number of ways. Therefore, the histo-architectural alterations of hepatopancreas are utilized as a sensitive, valuable, and rapid biomarker of xenobiotic-induced stress^68^. The histopathological effect of the two environmentally relevant concentrations of azoxystrobin on the tissue of the hepatopancreas in the land snail, *T. pisana* was examined. Regarding this, all histological observations in *T. pisana* showed that the tissue suffered histo-architectural harm upon treatment with the two tested concentrations of azoxystrobin.

While histopathological effects of many pesticides on tissues have been studied, azoxystrobin's effects on the digestive gland of terrestrial gastropods have not been investigated. Injuries to the hepatopencreas of snails caused by several pesticides have been documented by some researchers: Heiba et al.^69^ found that both species of snails, *Eobania vermiculata* and *Monacha contiana*, treated with the insecticide, methomyl had vacuolated and swollen digestive cells in association with many yellowish brown granules. Moreover, Hamed et al.^68^ showed histo-architectural alterations in the hepatopancreas of *E. vermiculata* subjected to methiocarb or methomyl using the baiting technique. Both pesticides resulted in significant cytoplasmic vacuolization and disruption with the lowering of microvilli, surface blab formation, an increase in the calcium spherule numbers in calcium cells, and an irregular increase in excretory cell numbers with a lot of excretory granules or residual bodies.

## Conclusions

The present study provides novel findings regarding exposure to ecologically relevant concentrations of azoxystrobin-based fungicides. According to our findings, animal growth and consumption of food decreased. The changes in antioxidant defense enzyme activities (CAT, GPx, and GST) and levels of LPO and GSH, indicating oxidative stress. Furthermore, the tested compound caused histopathological changes in the hepatopancreas of snails associated with physiological and biochemical alterations. Collectively, combining physiological, biochemical, and histopathological biomarkers into an integrated approach is a useful way to understand the real threat caused by azoxystrobin. Nevertheless, there is a need to identify other new and reliable biomarkers, such as omics, to provide more risk assessment information for ecosystems. Finally, our findings will draw attention to the adverse consequences of *T. pisana*, highlight its ecological risk to terrestrial ecosystems, and then find a way to mitigate the indiscriminate use of this pesticide.

## Data Availability

Data will be made available on request from the corresponding author.

## References

[1] Pathak VM (2022). Current status of pesticide effects on environment, human health and its eco-friendly management as bioremediation: A comprehensive review. Front. Microbiol..

[2] Zhang C (2020). Ecotoxicology of strobilurin fungicides. Sci. Total Environ..

[3] Bartlett DW (2001). Understanding the strobilurin fungicides. Outlooks Pest Manag..

[4] Kim JH (2007). Enhanced activity of strobilurin and fludioxonil by using berberine and phenolic compounds to target fungal antioxidative stress response. Lett. Appl. Microbiol..

[5] Zafar MI (2012). Ecological impacts of time-variable exposure regimes to the fungicide azoxystrobin on freshwater communities in outdoor microcosms. Ecotoxicology.

[6] USEPA, US-Environmental Protection Agency. Azoxystrobin pesticide fact sheet (1997).

[7] PPDB (Pesticide Properties Data Base), University of Hertfordshire. http://sitem.herts.ac.uk/aeru Accessed at 1 February 2023.

[8] Herrero-Hern’andez E, Marín-Benito JM, Andrades MS, S’anchez-Martín MJ, Rodríguez-Cruz MS (2015). Field versus laboratory experiments to evaluate the fate of azoxystrobin in an amended vineyard soil. J. Environ. Manag..

[9] Silva V (2019). Pesticide residues in European agricultural soils—A hidden reality unfolded. Sci. Total Environ..

[10] Hou Z, Wang X, Zhao X (2016). Dissipation rates and residues of fungicide azoxystrobin in ginseng and soil at two different cultivated regions in China. Environ. Monitor. Assess..

[11] EFSA. Conclusion on the peer review of the pesticide risk assessment of the active substance azoxystrobin. J. Eur. Food Saf. Author. EFSA Italy, **8**, 1542 (2010). 10.2903/j.efsa.2010.1872 PMC13085163 42004101

[12] Han YN (2014). Integrated assessment of oxidative stress and DNA damage in earthworms (*Eisenia fetida*) exposed to azoxystrobin. Ecotoxicol. Environ. Saf..

[13] Uçkun AA, Öz ÖB (2021). Evaluation of the acute toxic effect of azoxystrobin on non-target crayfish (*Astacus leptodactylus* Eschscholtz, 1823) by using oxidative stress enzymes, ATPases and cholinesterase as biomarkers. Drug Chem. Toxicol..

[14] El-Hak HNG, Al-Eisa RA, Ryad L, Halawa E, El-Shenawy NS (2022). Mechanisms and histopathological impacts of acetamiprid and azoxystrobin in male rats. Environ. Sci. Pollut. Res..

[15] Kovačević M, Stjepanović N, Hackenberger DK, Lončarić Ž, Hackenberger BK (2022). Toxicity of fungicide azoxystrobin to *Enchytraeus albidus*: Differences between the active ingredient and formulated product. Pest. Biochem. Physiol..

[16] Mesnage R, Antoniou MN (2018). Ignoring adjuvant toxicity falsifies the safety profile of commercial pesticides. Front. Public Health.

[17] Rozman KK, Doull J, Hayes WJ, Krieger R (2010). Dose and time determining, and other factors influencing, toxicity. Hayes' Handbook of Pesticide Toxicology.

[18] Van Gestel, C.A.M. Soil ecotoxicology: state of the art and future directions. In: Strus, J., Taiti, S., and Sfenthourakis, S. (Eds.), Advances in Terrestrial Isopod Biology, ZooKeys, **176**, 275–296(2012). 10.3897/zookeys.176.2275 PMC3335420 22536114

[19] Al-Alam J (2022). Snails as temporal biomonitors of the occurrence and distribution of pesticides in an apple orchard. Atmosphere.

[20] Gomot de Vaufleury A (2000). Standardized growth toxicity testing (Cu, Zn, Pb, and pentachlorophenol) with *Helix aspersa*. Ecotoxicol. Environ. Saf..

[21] Radwan MA, El-Gendy KS, Gad AF (2010). Biomarkers of oxidative stress in the land snail, *Theba pisana* for assessing ecotoxicological effects of urban metal pollution. Chemosphere.

[22] Louzon M, de Vaufleury A, Capelli N (2023). Ecogenotoxicity assessment with land snails: A mini-review. Mutation Res. Rev. Mutation Res..

[23] Davies KJ (2016). Adaptive homeostasis. Mol. Aspects Med..

[24] Marigomez IA (2013). Marine ecosystem health status assessment through integrative biomarker indices: a comparative study after the Prestige oil spill “mussel watch. Ecotoxicology.

[25] Vasseur P, Cossu-Leguille C (2003). Biomarkers and community indices as complementary tools for environmental safety. Environ. Int..

[26] Sogorb MA, Estevez J, Vitanova E, Gupta RC (2014). Biomarkers in Biomonitoring of Xenobiotics (Chapter 57). Biomarkers in Toxicology.

[27] Valavanidis A, Vlahogianni T, Dassenakis M, Scoullos M (2006). Molecular biomarkers of oxidative stress in aquatic organisms in relation to toxic environmental pollutants. Ecotoxicol. Environ. Saf..

[28] Radwan MA, El-Gendy KS, Gad AF (2020). Biomarker responses in terrestrial gastropods exposed to pollutants: A comprehensive review. Chemosphere.

[29] El-Gendy KS, Gad AF, Radwan MA (2021). Physiological and behavioral responses of land molluscs as biomarkers for pollution impact assessment: A review. Environ. Res..

[30] El-Gendy KS, Radwan MA, Gad AF (2011). Feeding and growth responses of the snail *Theba pisana* to dietary metal exposure. Arch. Environ. Contamin. Toxicol..

[31] Gomot A (1997). Effects of heavy metals on snail development. Use of snails as bio-indicators of heavy metal pollution for the preservation of human health. Bulletin de L’academie Nationale de Medecine.

[32] Placer ZA, Cushman LL, Jonson BC (1966). Estimation of product of lipid peroxidation (*Malondial Dehyde*) in biochemical systems. Anal. Biochem..

[33] Owens CWI, Belcher RV (1965). A colorimetric micro-method for the determination of glutathione. Biochem. J..

[34] Beers RF, Sizer IW (1952). Spectrophotometric method for measuring the breakdown of hydrogen peroxide by catalase. J. Biol. Chem..

[35] Chiu DTY, Stults FH, Tappel AL (1976). Purification and properties of rat lung soluble glutathione peroxidase. Biochimica et Biophysica Acta.

[36] Vessey DA, Boyer TD (1984). Differential activation and inhibition of different forms of rat liver glutathione S-transferase by the herbicides 2, 4-dichlorophenoxyacetate (2, 4-D) and 2, 4, 5-trichlorophenoxyacetate (2, 4, 5-T). Toxicol. Appl. Pharmacol..

[37] Ellman GL, Courtney KD, Andres V, Featherstone RM (1961). A new and rapid colorimetric determination of acetylcholinesterase activity. Biochem. Pharmacol..

[38] Lowry OH, Rosebrough NJ, Farr AL, Randall RJ (1951). Protein measurement with the folin phenol reagent. J. Biol. Chem..

[39] Banchroft JD, Stevens A, Turner DR (1996). Theory and Practice of Histological Techniques.

[40] Costat Program. Microcomputer program analysis. CoHort software, Version 2.6, Monterey, CA (2002).

[41] USEPA., United States-Environmental Protection Agency. US EPA-Pesticide; Azoxystrobin. Washington, D.C*. *20460. OPP Official Record. Health Effects Division (2010).

[42] Scott GR, Sloman KA (2004). The effects of environmental pollutants on complex fish behavior: Integrating behavioral and physiological indicators of toxicity. Aquat. Toxicol..

[43] McLoughlin N, Yin DQ, Maltby L, Wood RM, Yu HX (2000). Evaluation of sensitivity and specificity of two crustacean biochemical biomarkers. Environ. Toxicol. Chem..

[44] Chase R (2002). Behavior and Its Neural Control in Gastropod Molluscs.

[45] Slijkerman DME, Baird DJ, Conrad A, Jak RG, van Straalen NM (2004). Assessing structural and functional plankton responses to carbendazim toxicity. Environ. Toxicol. Chem..

[46] Schuytema GS, Nebeker AV, Griffis WL (1994). Effects of dietary exposure to forest pesticides on the brown garden snail *Helix aspersa* Müller. Arch. Environ. Contamin. Toxicol..

[47] El-Gendy KS, Radwan MA, Gad AF, Khamis AE, Eshra EH (2019). Physiological traits of land snails *Theba pisana* as simple endpoints to assess the exposure to some pollutants. Environ. Sci. Pollut. Res..

[48] Conti, M.E. *Biological Monitoring: Theory and Applications: Bioindicators and Biomarkers*. Technology and Engineering, pp. 228 (2008).

[49] Coeurdassier M, Saint-Denis M, Gomot de Vaufleury A, Ribera D, Badot PM (2001). The garden snail (*Helix aspersa*) as a bioindicator of organophosphorus exposure: effects of dimethoate on survival, growth, and acetylcholinesterase activity. Environ. Toxicol. Chem..

[50] Halliwell B, Gutteridge JMC (2007). Free Radicals in Biology and Medicine.

[51] Ali D, Ibrahim KE, Hussain SA, Abdel-Daim MM (2020). Role of ROS generation in acute genotoxicity of azoxystrobin fungicide on freshwater snail, *Lymnaea luteola* L. Environ. Sci. Pollut. Res..

[52] Prased SN, Muralidhara M (2012). Evidence of acrylamide induced oxidative stress and neurotoxicity in *Drosophila melanogaster*—Its amelioration with spice active enrichment: relevance to neuropathy. NeuroToxicology.

[53] Xu Y (2021). Ecotoxicity evaluation of azoxystrobin on *Eisenia fetida* in different soils. Environ. Res..

[54] Storey KB (1996). Oxidative stress: Animal adaptations in nature. Braz. J. Med. Biol. Res..

[55] Pen'a-Llopis S, Ferrando MD, Pena JB (2002). Impaired glutathione redox status is associated with decreased survival in two organophosphate-poisoned marine bivalves. Chemosphere.

[56] Canesi L, Viarengo A, Leonzio C, Filippelli M, Gallo G (1999). Heavy metals and glutathione metabolism in mussel tissues. Aquat. Toxicol..

[57] Vieira RSF, Venancio CAS, Felix LM (2021). Embryonic zebrafish response to a commercial formulation of azoxystrobin at environmental concentrations. Ecotoxicol. Environ. Saf..

[58] Lionetto MG (2003). Integrated use of biomarkers (acetylcholinesterase and antioxidant enzymes activities) in *Mytilus galloprovincialis* and *Mullus barbatus* in an Italian coastal marine area. Marine Pollut. Bull..

[59] Chandran R, Sivakumar AA, Mohandass S, Aruchami M (2005). Effect of cadmium and zinc on antioxidant enzyme activity in the gastropod, *Achatina fulica*. Comp. Biochem. Physiol. C.

[60] Hayes JD, Pulford DJ (1995). The glutathione S-transferase supergene family: Regulation of GST and the contribution of the isoenzymes to cancer chemoprotection and drug resistance. Crit. Rev. Biochem. Mol. Biol..

[61] Bhavan SP, Geraldine P (2000). Aberration in various parameters of bioenergetic in the *Macrobrachium malcolmsonii* following exposure to endosulfan. Aquaculture.

[62] Fulton MH, Key V (2001). Acetylcholinesterase inhibition in estuarine fish and invertebrates as an indicator of organophosphorus insecticide exposure and effects. Environ. Toxicol. Chem..

[63] Lionetto MG, Caricato R, Calisi A, Giordano ME, Schettino T (2013). Acetylcholinesterase as a biomarker in environmental and occupational medicine: new insights and future perspectives. BioMed Res. Int..

[64] Carageorgiou H, Tzotzes V, Sideris A, Zarros A, Tsakiris S (2005). Cadmium effects on brain acetylcholinesterase activity and antioxidant status of adult rats: Modulation by Zinc, Calcium and L-Cysteine Co-administration. Basic Clin. Pharmacol. Toxicol..

[65] Schallreuter KU, Gibbons NC, Elwary SM, Parkin SM, Wood JM (2007). Calcium- activated butyrylcholinesterase in human skin protects acetylcholinesterase against suicide inhibition by neurotoxic organophosphates. Biochem. Biophys. Res. Commun..

[66] Liu L, Zhu B, Wang GX (2015). Azoxystrobin-induced excessive reactive oxygen species (ROS) production and inhibition of photosynthesis in the unicellular green algae *Chlorella vulgaris*. Environ. Sci. Pollut. Res..

[67] Parvate YA, Thayil L (2017). Toxic effect of clove oil on the survival and histology of various tissues of pestiferous land snail *Achatina fulica* (Bowdich, 1822). J. Exp. Biol. Agric. Sci..

[68] Heiba FN, Al-Sharkawy IM, Al-Batal AA (2002). Effects of the insecticide, Lannate, on the land snails, *Eobania vermiculata* and *Monacha contiana*, under laboratory conditions. J. Biol. Sci..

[69] Hamed S, Abdelmeguied NE, Essawy AE, Radwan MA, Hegazy AE (2007). Histological and ultrastructural changes induced by two carbamate molluscicides on the digestive gland of *Eobania vermiculata*. J. Biol. Sci..

